# Clinical outcome, proteome kinetics and angiogenic factors in serum after thermoablation of colorectal liver metastases

**DOI:** 10.1186/1471-2407-13-266

**Published:** 2013-05-30

**Authors:** Marieke WJLAE Wertenbroek, Marianne Schepers, Hannetta J Kamminga-Rasker, Jan T Bottema, Anneke C Muller Kobold, Han Roelofsen, Koert P de Jong

**Affiliations:** 1 Department of Surgery, Division of Hepato-Pancreatico-Biliary Surgery and Liver Transplantation, University Medical Center Groningen, University of Groningen, PO Box 30 001, 9700 RB Groningen, the Netherlands; 2 Laboratory Medicine, University Medical Center Groningen, University of Groningen, PO Box 30 001, 9700 RB Groningen, the Netherlands; 3 Centre for Medical Biomics, University Medical Center Groningen, University of Groningen, PO Box 30 001, 9700 RB Groningen, The Netherlands

**Keywords:** Thermoablation, Proteomics, Angiogenesis, Liver tumor, Partial hepatectomy

## Abstract

**Background:**

Thermoablation is used to treat patients with unresectable colorectal liver metastases (CRLM). We analyze clinical outcome, proteome kinetics and angiogenic markers in patients treated by cryosurgical ablation (CSA) or radiofrequency ablation (RFA).

**Methods:**

205 patients underwent CSA (n = 20), RFA (n = 22), partial hepatectomy (PH, n = 134) or were found truly unresectable (n = 29). Clinical outcome, proteome transitions and angiogenic response in serum were analyzed at various time points after ablation.

**Result:**

Median overall survival in CSA patients (17.6 months) was worse (p < 0.0001) when compared to RFA treated patients (51.7 months) and patients after PH (43.4 months). The complication rate was higher in the CSA group (50%) as compared to the RFA group (22%). Proteomics analyses showed consistently more changes in serum protein abundance with CSA compared to RFA. In the first four days after ablation a pro-angiogenic serum response occurred.

**Conclusions:**

RFA of CRLM is superior to CSA with a median survival which equals survival in patients after PH. Proteomics analyses suggests a more aggravated serum response to CSA compared to RFA. Thermoablation is associated with changes in serum levels of angiogenic factors favouring a pro-angiogenic environment, but without differences between RFA and CSA.

## Background

Partial hepatectomy (PH) is a potential curative treatment for patients with colorectal liver metastases (CRLM). Unfortunately, the majority of patients are not amenable for PH because of bilobar metastases, widespread liver involvement or insufficient liver remnant. The poor prognosis of patients with unresectable CRLM resulted in the application of thermoablation either by cryosurgical ablation (CSA) or radiofrequency ablation (RFA). Although RFA seems to be the more widely applied technique, CSA is still in use in patients with CRLM or hepatocellular carcinoma (HCC) [1-6]. Since 2008, at least 12 original reports on 546 patients with liver tumors treated with CSA were published, of which 3 described more than 300 patients [6-8]. Contributing to the popularity of CSA might be the claimed anti-cancer immune response associated with cryoablation [9]. The results of RFA treatment for CRLM revealed 5-year survival rates of 14-55%, ablation site recurrence rates from 3.6 to 60%, and low major complication and mortality rates, 6-9% and < 2% respectively [10].

The question arises how these two ablation techniques compare to each other. In general one can conclude from the literature that the complication rate of CSA is higher than RFA [11,12]. This seems to be related to the systemic inflammatory response which is associated with CSA [13-15]. Several lines of evidence relate inflammation to the development and progression of cancer; angiogenic growth factors seem to be the common denominator for both conditions [16-18]. Especially angiopoietin-2 plays a dominant role not only in angiogenesis, but also in initiation and maintenance of inflammation [19-21]. A recent review summarized the relation between inflammation, angiogenesis and tumor progression and the possible impact that RFA of liver tumors could have [22].

The aim of the present study is to compare CSA versus RFA in patients with unresectable CRLM with respect to clinical outcome. Furthermore we wanted to evaluate the biochemical response and the temporal response in serum protein expression in both ablation groups. Finally we more specifically analyzed changes in the response of pro-angiogenic and anti-angiogenic growth factors in serum. We wanted to test the hypothesis that (1) CSA is associated with a more pronounced overall reaction with respect to protein kinetics and (2) CSA is associated with a more pronounced pro-angiogenic response as compared to RFA. To test these hypotheses we performed (1) a comprehensive approach to analyze the dynamics of the serum proteome and (2) analyzed changes in pro-angiogenic and anti-angiogenic serum molecules induced by CSA versus RFA in a subset of highly comparable patients in whom only ablation was performed and in whom no post ablation complications occurred.

## Methods

### Patient selection and treatment

The study is in compliance with the Declaration of Helsinki (Sixth Revision, 2008). The study fulfils all the requirements for patient anonymity and is in agreement with regulations of the Medical Ethics Committee of the University Medical Center Groningen for publication of patient data.

We used our prospectively maintained liver surgery database to identify all consecutive patients with CRLM who underwent a laparotomy with the intention to perform a PH alone or in combination with an ablation procedure. All patients underwent intentionally curative resections of the primary tumor, either previously or simultaneous with the liver procedure.

Patient treatment is according to a standard protocol, including CT-scan of thorax and abdomen, colonoscopy and CEA-serum level. If the CRLM were judged treatable by either PH or ablation or a combination of both, a laparotomy was performed. Ablation was performed if a PH alone was not able to render the liver tumor-free, except in 2 procedures in which comorbidity was the reason that ablation was conducted because these patients were not fit for PH. Suspicious lesions found during operation were sent for frozen section. Intraoperative liver ultrasound was done to rule out hitherto undetected CRLM. If the CRLM were judged resectable surgical treatment followed. If this situation could not be reached by PH and/ or ablation, the patients were defined as truly unresectable and included in the control (laparotomy only) group. The type of treatment was discussed in a multidisciplinary setting. No adjuvant chemotherapy was given postoperatively. Patients judged to be truly unresectable were offered the possibility of palliative chemotherapy. The Fong clinical risk score (CRS) was calculated [23].

### Equipment used for tumor ablation

Ablation was performed under intra-operative ultrasound guidance according to the manufacturers’ protocol. Cryoablation was performed under using the Cryo 6 equipment (Erbokryo-CS6 equipment, ERBE, Tübingen, Germany). The RF 3000 TM Radio Frequency Ablation System (Boston Scientific, Boston, MA, USA) was used for RFA.

### Follow-up of patients

All patients had the potential of at least 5 years of follow-up. Mortality was defined as any death during hospitalization or within 30 days from surgery. Follow-up was performed every 3 months during the first two years and every 6 months thereafter and included serum CEA, liver ultrasound and thoracic X-ray in patients after PH. Suspicious lesions were confirmed by CT scan or magnetic resonance imaging (MRI) in case of contrast allergy. Because CT scanning of ablated tumors is the preferred imaging modality, CT (or MRI) replaced ultrasound in patients treated with ablation [24]. Recurrences were treated by resection or ablation if limited or with chemotherapy if local treatment was not intentionally curative.

### Biochemical and angiogenic growth factor serum response after CSA or RFA

We selected 12 patients (n = 6 CSA, n = 6 RFA) with comparable clinicopathological characteristics who underwent ablation as a sole treatment –without concomitant PH- and who had an uncomplicated postoperative course. These patients were used for comparison of the response of relevant serum markers, serum protein expression and angiogenic growth factors during the first 4 days after operation. None of these patients received any transfusions of blood or thrombocytes or plasma products. Routine blood tests are performed in all patients admitted for liver surgery and serum samples were obtained by venipuncture at the day of admission to the hospital (−1) at the end of surgery (day 0, only proteomics and angiogenic growth factors) and at days 1, 2, 3 and 4. We considered the following markers relevant; CRP and albumin as markers of the acute phase response, LDH and ALAT to quantify liver tissue damage, thrombocytes as a reservoir of angiogenesis-related molecules and antithrombin III (AT III) because of its anticoagulant, anti-inflammatory and anti-angiogenic capacity. The following angiogenic molecules were determined: vascular endothelial growth factor (VEGF), hepatocyte growth factor (HGF), angiopoietin-2 (Ang-2), human Tie-2 (all Quantikine ELISA kits, R&D Systems, Minneapolis, USA). The anti-angiogenic molecules angiopoietin-1 (Ang-1), endostatin (both Quantikine ELISA kits) and angiostatin (RayBiotech Inc, Norcross, USA) were determined. Values were expressed as a percentage change of the patients’ growth factor concentrations at the day of admission (day −1).

### SELDI-TOF-MS analysis

Monitoring the time-dependent protein expression dynamics that take place during disease progression, recovery from surgery or in response to treatment or diet may help to understand the underlying physiological and biochemical processes as we have previously shown [25]. SELDI-TOF-MS is a high-throughput proteomics technology that allows rapid acquisition of protein expression profiles however it does not allow identification of individual proteins but can show biological processes that involve changes in serum protein abundance. Serum samples were processed as described previously [25]. Briefly, samples were denatured by mixing 600 μl sample with 400 μl 20% v/v acetronitril. Half of the denatured sample was used directly for SELDI measurement with sinapinic acid (SPA) as matrix. The other 500 μl was used to prepare a low molecular weight fraction using an ultra-filtration step with a 50 kDa cut-off and was measured with α-cyano-4-hydroxycinnamic acid (CHCA) as matrix. Samples were randomly applied in triplicate to CM10 ProteinChip arrays and measured in a ProteinChip system 4000 mass spectrometer (both from Bio-Rad, Hercules CA, USA). Ciphergen Express software 3.0 was used for data analyses with default settings for baseline removal and normalization for total ion current. Peaks that had a signal to noise ratio ≥ 5 in at least 3 spectra were clustered (mass deviation ≤ 0.3%). In the final dataset peaks detected with CHCA, in the mass ranges 1–6.5 kDa and 6.5-30 kDa, were combined with peaks detected with SPA, in the mass range 2–100 kDa.

### Statistics

Continuous variables are presented as median and interquartile range (IQR). Survival after surgery was calculated from the day of surgery until the last follow-up date (May 2011) or until the day of death using the Kaplan-Meier method with the log-rank test for comparison. Because patient inclusion stopped at May 2006, all patients had the potential of at least 5 years of follow-up and therefore actual survival data are presented. None of the included patients were lost to follow-up except for deceased patients. Factors associated with overall and disease-free survival were examined using univariate and multivariate Cox regression analysis. Chi square test was used for comparison of categorical variables. The serum response was compared using baseline (day −1) values, and by measuring the total response as area under the curve (AUC) from time point 0 (day of operation) to day 4. Comparisons were done using the Mann–Whitney U test. All p-values were derived from two-tailed tests and were considered significant if < 0.05.

## Results

### Clinical outcome of all patients

#### Patient and tumor characteristics in the four patient groups

In total 205 patients were treated for CRLM. In 134 patients a PH was performed and 29 patients were found truly unresectable. In 42 patients local ablation with CSA (n = 20, of which 7 in combination with PH) or RFA (n = 22, of which 11 in combination with PH) was performed. Baseline patient and tumor characteristics were not different in the CSA, RFA, PH and truly unresectable groups except for the number of metastases, CRS and surgical procedure (Table 1). In the RFA and truly unresectable groups about 68% (35/47) of the patients had more than one metastasis whereas in the PH group 64% (86/134) of patients had a solitary metastasis. Patients in the RFA group had a higher CRS as compared to the other groups.

**Table 1:** Baseline patient and tumor characteristics

**Clinicopathological characteristics**	**CSA**	**RFA**	**PH**	**Unresectable**	***p-*value**
n	20	22	134	29	
Median age (years)	66.7	60.0	62.3	60.8	0.151
(IQR)	(9.3)	(16.7)	(13.8)	(17.4)	
Sex					0.392
Female	10 (50%)	10 (45%)	55 (41%)	8 (28%)	
Male	10 (50%)	12 (55%)	79 (59%)	21(72%)	
Site of primary tumor					0.732
Colon	12 (60%)	15 (68%)	84 (63%)	21(72%)	
Rectum	8 (40%)	7 (32%)	50 (37%)	8 (28%)	
Synchronous: metachronous liver metastasis	4:16	10:12	44:90	14:15	0.136
Interval resection of primary tumor and detection liver metastasis					0.385
≤12 months	9 (45%)	14 (64%)	88 (66%)	20 (69%)	
>12 months	11(55%)	8 (36%)	46 (34%)	9 (31%)	
Node status of primary tumor					0.479
Negative	8 (40%)	6 (27%)	50 (37%)	7 (24%)	
Positive	12 (60%)	16 (63%)	84 (63%)	22 (76%)	
Adjuvant chemotherapy after primary					0.470
No	12 (60%)	12 (55%)	92 (69%)	21 (72%)	
Yes	8 (40%)	10 (45%)	42 (31%)	8 (28%)	
Size largest metastasis (cm)					
Median (IQR)	4.0 (2.0)	3.0 (2.0)	5.0 (4.5)	3.5 (4.0)^*^	0.440
Number of liver metastases					<0.0001
1	10 (50%)	6 (27%)	86 (64%)	6 (21%)	
>1	10 (50%)	16 (63%)	48 (36%)	19 (66%) ^γ^	
Preoperative CEA (μg/L)					
Median (IQR)	21.2 (64.8)	12.0 (57.0)	22.5(71.4)^$^	20.0 (116.3)^&^	0.623
CRS					0.009
≤2	15(75%)	10 (46%)	89 (66%)		
>2	5 (25%)	12 (55%)	45 (34%)		
Surgical procedure					<0.0001
No resection	13 (65%)	11 (50%)	-	29 (100%)	
< Hemihepatectomy	6 (30%)	7 (32%)	36 (27%)		
Hemihepatectomy	1 (5%)	3 (14%)	56 (42%)		
Extended	-	1 (5%)	42 (31%)		
Hemihepatectomy					

*5 missing.

^γ^ 4 missing.

^$^ 9 missing.

^&^ 2 missing.

*CSA*, Cryosurgical ablation.

*RFA*, Radiofrequency ablation.

*PH*, Partial hepatectomy.

*CEA*, Carcinoembryonic antigen.

*CRS*, Clinical risk score.

The indications for performing local ablation in the 42 patients were comorbidity precluding PH (n = 1, each ablation group), bilobar disease with PH of one hemiliver and unresectable liver tumors in the other hemiliver (n = 6 CSA, n = 11 RFA), expected insufficient future liver remnant (n = 10 CSA, n = 6 RFA) and inability to perform a PH because of a unresectable deeply located recurrence after previous hemihepatectomy (n = 1 CSA), localization in liver (n = 1 RFA), minimal residual disease after chemotherapy (n = 2 CSA), or simultaneous resection of colon in patients in whom a combination of hemihepatectomy and primary tumor resection was considered not feasible (n = 3 RFA). In patients with solitary liver metastasis (n = 14) ablation was in the majority conducted because of an expected insufficient future liver remnant. In a single case, the reason was the inability to perform a PH because of a non-resectable deeply located recurrence after previous hemihepatectomy, comorbidity precluding PH or minimal residual disease after chemotherapy.

#### Complication rate

No postoperative mortality occurred after ablation. In the PH group 6 out of 134 patients (4.5%) died within 30 days after surgery. Three postoperative deaths were caused by liver failure and three patients died of pulmonary complications. Complications occurred in 5/22 RFA patients (23%) and 10/20 CSA patients (50%, p = 0.06). More severe complications like iceball cracks (n = 2), biliary problems (n = 3) and kidney failure (n = 2) were only present in CSA patients.

#### Patterns of tumor recurrence and survival

In 106 of 176 patients (60%) recurrences developed (Table 2). The rate of recurrences was higher in CSA patients (90%, 18/20) and RFA patients (73%, 16/22) than in the PH group (54%, 72/134, p = 0.004). Recurrences were at multiple sites in 12/20 CSA patients and in 3/22 RFA patients (p = 0.005). Ablation site recurrences were seen in 9/20 CSA patients (45%) and in 4/16 RFA patients (18.2%, p = 0.096). In 7 of the 9 CSA treated patients with ablation site recurrences more disseminated recurrent disease was present. In RFA treated patients only 1 out of 4 ablation site recurrences was part of more disseminated recurrences. In 5/16 RFA treated patients with recurrences a liver directed second procedure could be performed because the recurrences were confined to the liver (ablation site: n = 2, liver remnant: n = 2, or both: n = 1). In the CSA group 3/18 patients with recurrences underwent a reoperation; two because of an ablation site recurrence and one because of a recurrence in the liver remnant.

**Table 2:** Patterns of recurrence and survival after cryosurgical ablation (CSA), radiofrequency ablation (RFA) or partial hepatectomy (PH) of colorectal liver metastases

	**CSA**	**RFA**	**PH**
	**(n = 20)**	**(n = 22)**	**(n = 134)**
**No recurrences**	2	6	62
**Recurrences**	18	16	72
*Single site*	6	13	45
Abdomen (extrahepatic)	1	4	8
Liver remnant	2	5	12
Ablation site	2	3	
Lung/thorax	1	1	20
Other single sites	-	-	5
*Multiple sites*	12	3	27
*Ablation site recurrences*	9	4	
- ablation site only	2	3	
- ablation site and other sites	7	1	
*Median interval to recurrence* (months)	8.5	9.0	9.0
(Range)	(3–44)	(2–31)	(3–72)
Median overall survival (months)	17.6	51.7	43.4
5-year overall survival rate (%)	5	38	42

**Figure 1:**
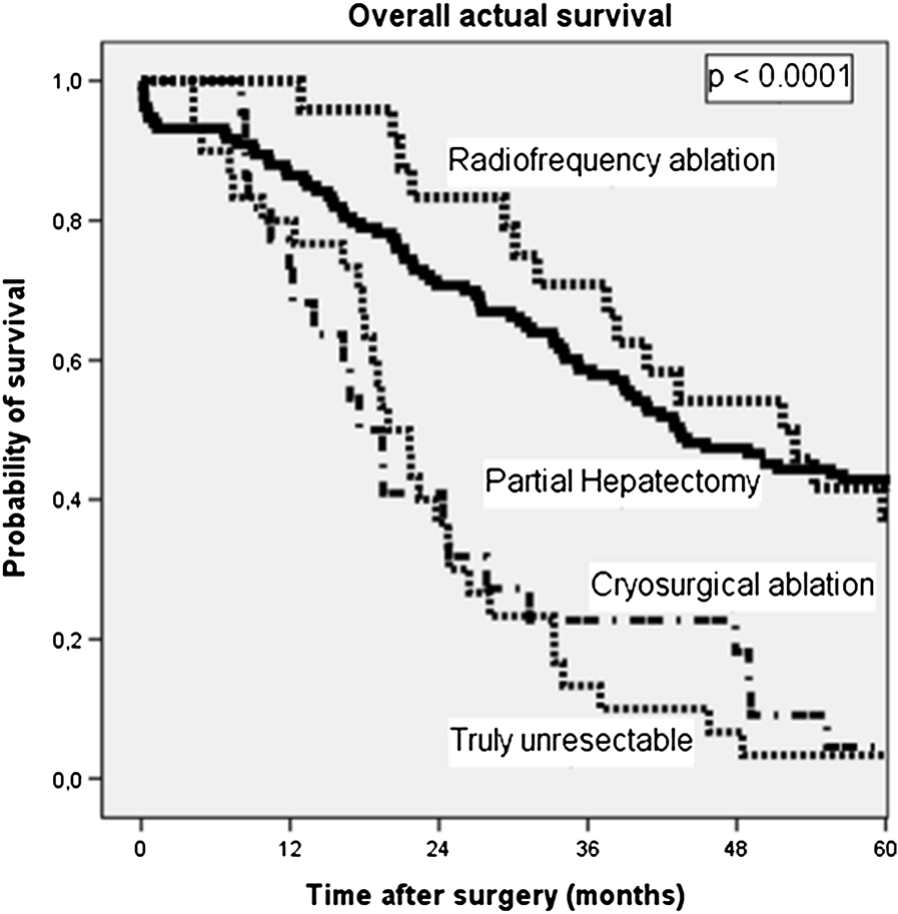
**Kaplan-Meier survival plot of overall survival in patients with colorectal liver metastases according to treatment.** Partial hepatectomy includes patients treated only by partial hepatectomy. Thermoablation includes patients treated with thermoablation with or without partial hepatectomy.

Overall actual survival for the CSA, RFA, PH and truly unresectable group are shown in Figure 1. The median survival of patients treated with RFA (51.7 months) or PH (43.4 months) was longer (p < 0.0001) than the median survival of both the CSA treated group (17.6 months) and the truly unresectable group (19.9 months). Results of multivariate analysis using Cox regression, showing the relative risk of dying (overall survival) and relative risk of recurrence (disease-free survival) and 95% confidence interval (CI) compared to the reference standard partial hepatectomy (PH, 1.00) are shown in Table 3.

**Table 3:** The relative risk of dying, recurrence and 95% confidence interval compared to the partial hepatectomy

**Prognostic factor**	**Risk of dying**			**Risk of recurrence**		
	**Relative risk**	**95% CI**	**P**	**Relative risk**	**95% CI**	**P**
**Surgical procedure**			<0.0001			<0.0001
PH	1.00			1.00		
Truly unresectable	2.63	1.43-4.82	0.002	2.63	1.43-4.82	0.002
CSA	3.27	1.99-5.37	<0.0001	3.12	1.91-5.11	<0.0001
RFA	0.84	0.49-1.45	0.54	0.84	0.50-1.43	0.52
**Clinical risk score**			0.002			0.002
0	1.00			1.00		
1	3.14	0.96-10.3	0.059	2.50	0.88-7.09	0.085
2	2.72	0.84-8.84	0.097	2.26	0.81-6.33	0.12
3	4.81	1.47-15.78	0.01	3.81	1.34-10.80	0.012
4	6.66	1.94-22.92	0.003	5.53	1.86-16.49	0.002
5	17.65	1.71-182.66	0.016	14.17	1.48-136.21	0.022

*CI*, Confidence interval.

*PH*, Partial hepatectomy.

*CSA*, Cryosurgical ablation.

*RFA*, Radiofrequency ablation.

On multivariate analysis the type of surgical procedure and the clinical risk score were independent prognostic variables both for overall survival and disease-free survival. Of note, on multivariate analysis, the relative risk of dying or developing recurrent disease after RFA was not different (RR: 0.84; 95% CI: 0.50-1.45, p > 0.50) as compared to the reference group of patients after PH.

### Response in the subset of patients treated with ablation alone

#### Baseline patient and tumor characteristics

A comparison of the patients solely treated with open ablation revealed no differences in age (median (IQR): CSA 70 (9.4), RFA 60 years (22.7)), size of treated metastasis (median (IQR): CSA 4.5 (3.0) cm, RFA 5.5 (4.3) cm), number of metastases 1:>1 (CSA 4:2, RFA 2:4), median (IQR) CRS (CSA 1.5 (1.5), RFA 2.5 (1.3)), or operation time (median (IQR) CSA 408 (92.5), RFA 320 (152.5) minutes), all p >0.05.

#### Proteomics analyses of serum response after CSA versus RFA

**Figure 2:**
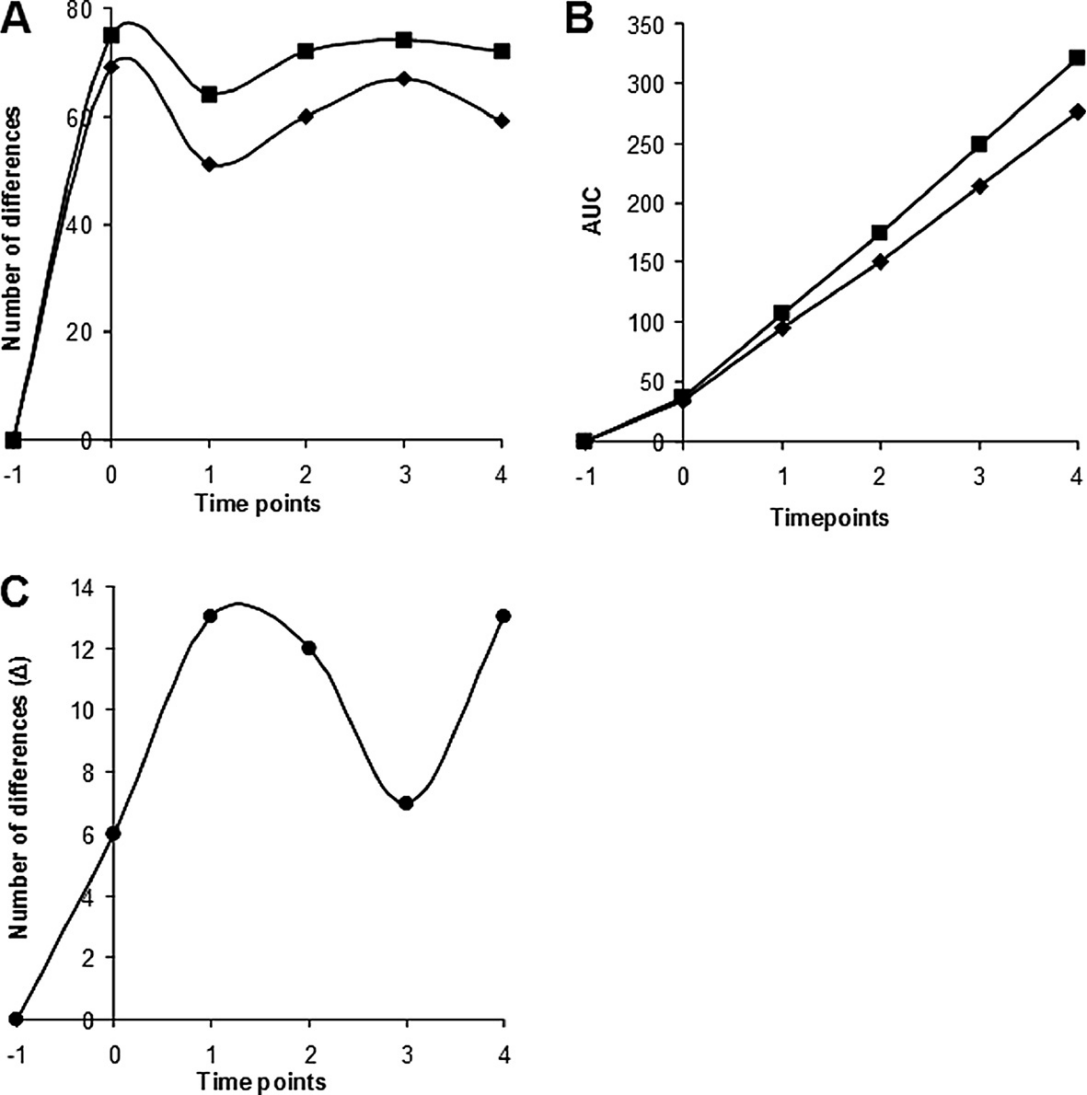
**SELDI-TOF-MS proteomics time series analyses.** Proteomics analyses of differences in serum protein abundance in samples obtained from six patients treated with radiofrequency ablation (RFA: ♦) and six patients treated with cryosurgical ablation (CSA: ■). Panel **A**: number of differences for RFA and CSA compared to time point −1 (before surgery). Panel **B**: AUC is higher at time points 1 through 4 for CSA than for RFA. Panel **C**: number of differences between RFA and CSA. Time −1 = day of admission; 0 = at the end of operation; 1–4 = postoperative days 1–4.

As shown in Figure 2A, ablation induced a biphasic pattern with more than 70 changes in protein abundance in serum at time point 0 (Figure 2 panel A). At this time point as well as all subsequent time points more changes were observed with CSA than with RFA as is also evident from the AUC (Figure 2 panel B). At time points 1, 2 and 4 the largest differences were found (Figure 2 panel C).

#### Biochemical and angiogenic growth factor serum response after CSA versus RFA

**Figure 3:**
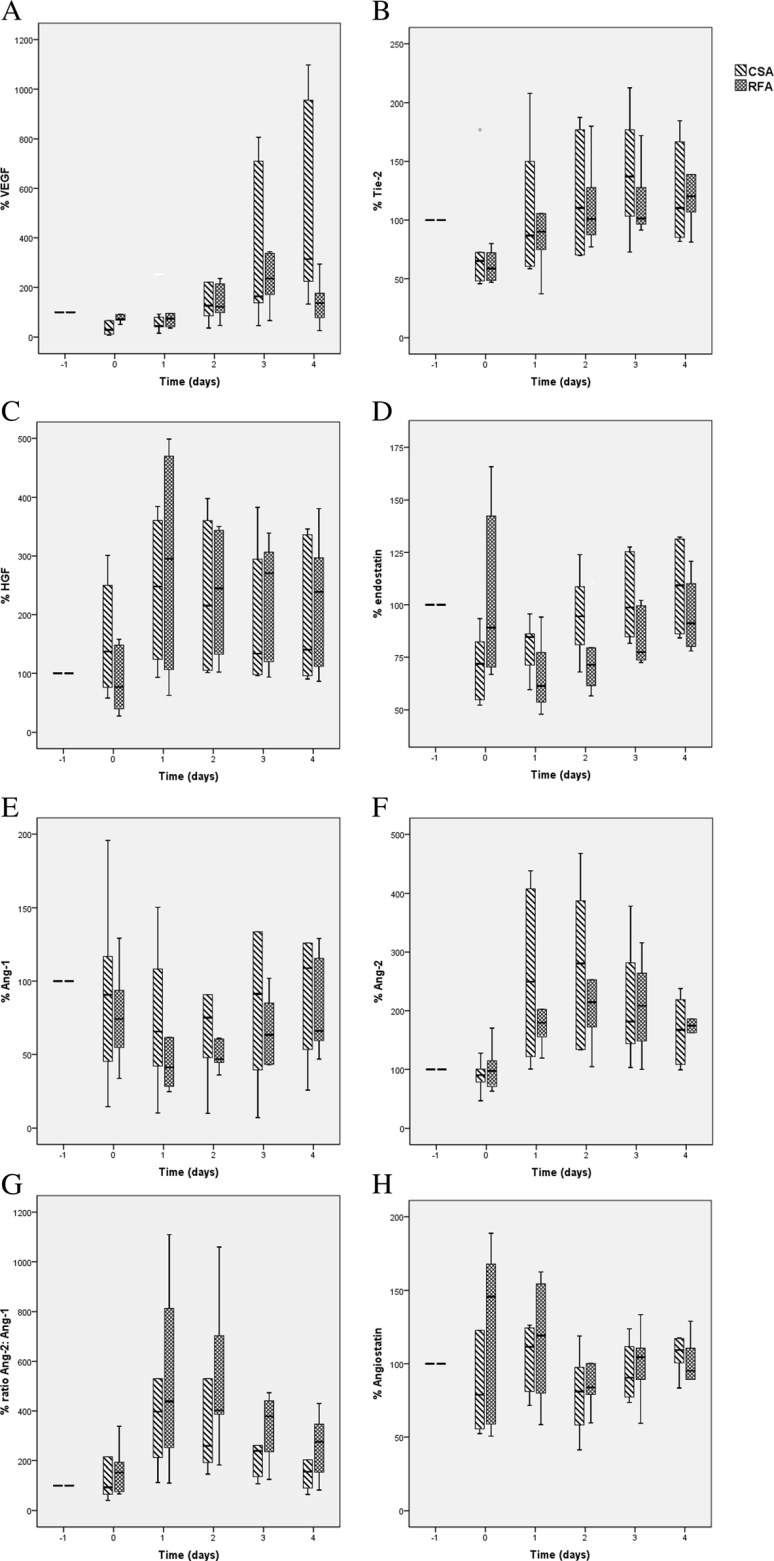
**Percentual change in angiogenic factors in patients treated with open ablation (CSA**** versus RFA****).** Panel **A**: VEGF, **B**: Tie-2, **C**: HGF, **D**: endostatin, **E**: Ang-1, **F**: Ang-2, **G**: Ratio Ang-2: Ang-1, **H**: Angiostatin. Thermoablation is associated with a pro-angiogenic response as reflected by an increase in serum levels of pro-angiogenic molecules (VEGF, Tie-2, Ang-2 and HGF). The response is comparable in patients with CSA and RFA treated tumors. Baseline values at time point −1 are set at 100%. Time −1 = day of admission; 0 = at the end of operation; 1–4 = postoperative days 1–4.

Baseline values at day −1 of biochemical and angiogenic growth factors were not different in the RFA versus the CSA group (data not shown). The total response of serum CRP, albumin, LDH, ALAT, thrombocyte count and AT II was not different except for LDH which was higher in the CSA group versus the RFA group (p = 0.02, data no shown). The median (IQR) baseline values for VEGF were 270.0 (308.9) pg/mL, HGF: 1,529.0 (872.4) pg/mL, Ang-1: 27,719.5 (22,635) pg/mL, Ang-2: 1,988.3 (1,107.0) pg/mL, endostatin: 136.1 (47.8) ng/mL, Angiostatin: 53.1 (73.0) ng/mL, and for Tie-2: 28.0 (12.0) ng/mL. The percentual change of the serum levels of the various angiogenic factors is presented in Figure 3. All molecules playing a role in the pro-angiogenic response (VEGF, Tie-2, Ang-2 and HGF) demonstrate an increase in serum levels after ablation. Ang-2 and HGF show an immediate postoperative increase whereas VEGF serum levels start to rise only after day 1. Tie-2 levels demonstrate an initial decline before they start rising above baseline levels after day 1. The ratio Ang-2/Ang-1 increases sharply after operation with an increase to >400% at day 1 after operation, which stays high the second day for RFA. The vessel stabilizing factor Ang-1 shows a decline after ablation. The inhibitors of angiogenesis, endostatin and angiostatin show a slightly different response. Endostatin levels reveal a postoperative decline with restoration to baseline levels at day 4. Angiostatin levels demonstrate a 40% increase at the end of the operation with a decline afterwards. A comparison of the AUC for the angiogenic molecules did not reveal differences (p >0.310) between CSA versus RFA.

## Discussion

For patients with unresectable CRLM thermoablation is a potential curative option, and both RFA and CSA are applied [26,27]. The aim of the present study was to compare the clinical outcome in patients with CRLM treated with CSA versus those treated with RFA and to more specifically analyze temporal changes in serum proteome expression as well as molecules relevant for the pro-angiogenic and anti-angiogenic balance in patients treated with CSA or RFA.

We found that CSA does not contribute to prolonged survival because the median overall survival of patients treated with CSA is as poor as truly unresectable patients. In contrast, the median overall survival of patients treated with RFA (51.7 months) is comparable to that of patients treated by PH (43.4 months). Remarkably, this comparable survival is despite a negative selection of RFA patients, as reflected by a higher clinical risk score as compared to PH patients. We also found that recurrences after CSA are more often at multiple sites as compared to recurrences after RFA, which are more often at single -and thus potentially surgically treatable- sites. A possible explanation for this is that CSA has more profound systemic effects than RFA, which, if it is associated with a sepsis-like condition, is known as the “cryoshock phenomenon”. Our proteomics analyses of serum protein dynamics indeed showed a biphasic pattern with consistently more changes in serum protein abundance with CSA compared to RFA at all-time points measured. This biphasic pattern is similar to that observed in a previous study [25] on the kinetics of the serum proteome after colon surgery and reminiscent of an acute phase response that appears more severe with CSA than with RFA.

This indicates that the response to CSA is more aggravated and thereby creates an environment in which circulating tumor cells are more likely to adhere to activated endothelial cells in remote organs and tissues and thus contribute to tumor seeding of micrometastases. Also in rodents an augmented systemic inflammatory response has been demonstrated after CSA [15,28].

Based on the proteomics results, we subsequently tried to identify whether angiogenic proteins are part of this protein abundance after CSA. These angiogenic proteins could probably initiate the angiogenic switch resulting in progression of non-angiogenic micrometastases into clinically detectable tumor masses [29,30]. Therefore we hypothesized that CSA is associated with a more pronounced pro-angiogenic serum response than RFA. Based on an exploratory analysis of pro-angiogenic and anti-angiogenic molecules we conclude that thermoablation of CRLM is associated with a pro-angiogenic response, but that the angiogenic serum response was comparable in CSA and RFA patients; the time course analysis after thermoablation of all investigated angiogenic molecules revealed no statistically significant differences. Current knowledge with respect to angiogenesis states that Ang-1, which is widely expressed in human tissues, constitutively activates Tie-2, which is almost exclusively expressed in endothelial cells, and thereby maintains blood vessel [21,31]. Ang-2 is mainly produced by endothelial cells and is active at sites of vascular remodeling both in physiological and pathological conditions. Upregulation of Ang-2 results in binding to Tie-2 and destabilization of vessels, which in the presence of pro-angiogenic cytokines, like VEGF, will result in angiogenesis, whereas in the absence of pro-angiogenic activity vessel regression will follow. Especially relevant in this respect is the Ang-2/Ang-1 ratio, which is often increased in tumors and its surroundings, which suggests that the angiogenic balance is tipped towards a pro-angiogenic state [32-34]. Also in the present study we found a four-fold increase in the Ang-2/Ang-1 ratio at day 1 and a slow decrease afterwards but still a two to three-fold higher ratio at day 4 after the ablation. Interestingly, Ang-2 also potentiates the effect of inflammatory cytokines by induction of expression of several adhesion molecules [35]. This tight relation between the inflammatory response and Ang-2 serum levels is exemplified in our study by the similar course of Ang-2 serum levels and CRP levels, both of which have their peak values at day 2 after ablation. It is of interest to evaluate whether the magnitude of the procedure is of influence on the levels of these growth factors. In one study in patients after partial hepatectomy substantially higher HGF and VEGF serum levels were found as compared to our data, suggesting a correlation between the extent of the procedure [36].

Median survival and overall 5-year survival after RFA and PH are comparable to those in a recently published large series of predominantly resected patients [37].

What is the evidence that thermodestruction by heat (RFA) has different pathophysiological effects as compared to ablation by cold (cryoablation)? The mechanisms through which cryoablation induces cell injury have recently been reviewed [38]. Gage et al. nicely summarizes literature evaluating the lowest temperature which should be reached to induce tissue damage –the lethal temperature-; it shows that this is highly variable in various tissues and organs. One of the most potent effects of thermoablation is derangement of protein stability. Remarkably, these effects have been less intensely studied during freezing as compared to during heating [39]. In a study in mice subjected to cold stress it was demonstrated that induction of heat shock proteins –responsible for protection of cells to stress- is different in hypothermia versus hyperthermia [40]. Also, in a rat model of ventilator-induced lung injury, plasma levels of pro-inflammatory cytokines were highest in hyperthermic rats as compared to hypothermic rats; the reverse holds true for the anti-inflammatory interleukin-10 [41].In vitro experiments using human white blood cells stimulated by lipopolysaccharide at various temperatures revealed a temperature dependent dysregulation of several microRNAs regulating cytokine production [42]. The differences between the responses to hyperthermia versus hypothermia were very nicely demonstrated in a study using microarray analysis of gene expression in a human hepatocellular carcinoma cell line [43]. The authors were able to demonstrate that hypothermia induces changes in relative expression of 409 gene expression sequences, whereas hyperthermia induces changes in “only” 71 sequences. Burattini et al. [44] reviewed the response patterns of cells under the influence of various physical agents. They summarized that mild heat exposure of cell cultures mainly induces cell apoptosis which is in contrast to hypothermia which rarely induces apoptosis but more frequently necrosis. All these data suggest that the pathophysiological response to thermoinjury is variable and dependent on the temperature changes. Of note, the above mentioned papers all study temperature changes in the physiological range whereas cryoablation or radiofrequency ablation creates temperatures in the supranormal or subnormal range of temperatures. Further evaluation of the response to RFA or cryoablation in humans with liver tumors is warranted to more clearly elucidate the pathophysiological responses in relation to dissemination and growth of tumor cells.

A possible limitation of our study could be that the fast development of the ablation equipment has resulted in more effective ablation devices nowadays. However, we think that it is the low temperature which destroys the tumor and additionally an adequate margin around the tumor dictates the success of the treatment. As long as these two prerequisites are fulfilled the type of device which creates the ice ball is, to our opinion, less of importance. Another limitation of the study is the small number of patients in whom the analysis of proteomic dynamics and angiogenic molecules was performed. The patients in both groups were however highly comparable with respect to baseline characteristics, treatment (solely by ablation), transfusion requirements (none) and postoperative complications (none). All of these can influence the results of serum protein profiles and angiogenic molecules. Another strong aspect of this study is that it is the first which investigates a set of angiogenic markers instead of just one relevant angiogenic factor, for instance VEGF, as is often done in the literature. Using this approach we were able to measure an integral response of key molecules relevant in the balance of pro-angiogenesis and anti-angiogenesis. Despite these limitations our study has advantages in that it is a monocentric study and patients were treated in a protocolled manner. Another important advantage of the present study is the strict and closed follow-up with none of the 205 patients lost to follow-up for other reasons than death. Moreover, because all patients were included before May 2006 they all had a potential minimal follow-up of 5 years. Therefore the survival analysis of all patients is based on actual data instead of estimations which form the basis for actuarial survival. Censored patients (those with missing information on survival time) can influence results, for instance by overestimation of survival [45,46].

## Conclusions

We have shown that in patients with unresectable CRLM, RFA significantly improves survival, even up to the same survival as is obtained with PH. In contrast, CSA does not contribute to a better survival and is associated with a higher complication rate. Although –based on the proteome analysis- we found that CSA initiates a more profound response we could not find a difference in biochemical or angiogenic response. Ablation generates changes in circulating angiogenic factors favoring a pro-angiogenic environment. Further studies are needed to examine which proteins are contributing to the more pronounced effect on protein kinetics in CSA treated patients.

## Abbreviations

PH: Partial hepatectomy; CRLM: Colorectal liver metastases; CSA: Cryosurgical ablation; RFA: Radiofrequency ablation; HCC: Hepatocellular carcinoma; CRS: Clinical risk score; MRI: Magnetic resonance imaging; AT III: Antithrombin III; VEGF: Vascular endothelial growth factor; HGF: Hepatocyte growth factor; Ang-1: Angiopoietin-1; Ang-2: Angiopoietin-2; SPA: Sinapinic acid; CHCA: α-Cyano-4-hydroxycinnamic acid; IQR: Interquartile range; AUC: Area under the curve.

## Competing interests

The authors declare that they have no competing interests.

## Authors’ contributions

MWJLAEW, participated in the sequence alignment, drafted the manuscript, participated in the design of the study and performed the statistical analysis. MS, HJK-R, JTB and ACMK carried out the immunoassays. HR carried out the immunoassays, participated in the design of the study and performed the statistical analysis. KPdJ conceived of the study, participated in its design and coordination, helped to draft the manuscript, and performed the statistical analysis. All authors read and approved the final manuscript.

## Pre-publication history

The pre-publication history for this paper can be accessed here:

http://www.biomedcentral.com/1471-2407/13/266/prepub
